# Population dynamics of *Meloidogyne graminicola* in soil in different types of direct-seeded rice agroecosystems in Hunan Province, China

**DOI:** 10.2478/jofnem-2023-0040

**Published:** 2023-12-31

**Authors:** Zhuhong Yang, Lu Zhang, Xinwen Li, Yufeng Lin, Shan Ye, Zhong Ding

**Affiliations:** College of Plant Protection, Hunan Agricultural University, Changsha 410128, China; Hunan Provincial Engineering and Technology Research Center for Biopesticide and Formulation Processing, Changsha, 410128, China; Agriculture and Rural Department of Hunan Province, Plant Protection and Inspection Station, Changsha 410005, China

**Keywords:** *Meloidogyne graminicola*, population density, agroecosystem, soil moisture, root gall

## Abstract

The rice root-knot nematode *Meloidogyne graminicola* is increasingly widely distributed in China and has had a severe incidence in Hunan Province. It is thus necessary to investigate its population dynamics in paddy fields. This study was conducted to ascertain the effect of direct-seeded rice agroecosystems on the population dynamics of *M. graminicola* and root gall development in rice. The results indicated that the population density of *M. graminicola* in soil was markedly influenced by the agroecosystem, rainfall and temperature. The population density of *M. graminicola* J2, and eggs in the soil and root galls, were significantly larger in the dry aerobic rice agroecosystem and in the rain-fed upland agroecosystem than in the lowland double-rice cropping sequence agroecosystem. As it can affect soil moisture rainfall was the key factor affecting the density of nematodes in both the rain-fed upland agroecosystem and the dry aerobic rice agroecosystem. Field flooding was still an effective way to reduce the population density of *M. graminicola*. In addition, we observed that *M. graminicola* can lay eggs outside rice roots under laboratory conditions. Therefore, we propose a hypothesis that *M. graminicola* lays egg masses within roots when the soil moisture is high, but lays eggs outside when the soil moisture is suitable. By clarifying the population dynamics of *M. graminicola* in different types of direct-seeded rice agroecosystems, this study is conducive to controlling rice root-knot nematodes.

## Introduction

Rice is an important staple food for a large part of the world’s population. Asia accounts for almost 90% of global rice production, with nine of the top ten rice producing and consuming countries in Asia (Surendran et al., 2021). As a major rice-production country, China contains approximately 18.2% of the world’s rice planting area and accounts for 27.2% of the world’s rice output (FAO, 2021). Traditional transplant irrigation is the main production system for rice, and nearly 95% of rice is grown under such long-term flooded conditions in China. Southern provinces can grow two or three rice crops in a year, but northern regions can only grow single-season rice (Peng et al., 2009).

However, the increase in extreme heat and drought events, combined with labor shortages and water shortages for agricultural irrigation, have led to the adjustment of the structure of the agricultural industry in China (Lu et al., 2016; Hanjra & Qureshi, 2010). To address these challenges, simplified cultivation techniques such as direct seeding and throwing seedlings have become increasingly attractive in rice crop production due to requiring less water and less labor (Liu et al., 2015; Huang et al., 2011).

Direct-seeding rice involves sowing pregerminated seeds in puddled soil (wet sowing), standing water (water sowing), or dry seeding on a prepared seedbed (dry sowing). However, the transition to direct-seeding methods brings forth significant challenges related to weeds, diseases and insect pests. Moreover, altering rice cultivation practices impacts both rice ecosystems and the rice rhizosphere community (Chaudhary et al., 2023; De Waele & Elsen, 2007; Farooq et al., 2011).

*Meloidogyne graminicola*, commonly referred to as the rice root-knot nematode, is considered a major threat to rice because it causes severe rice yield losses, particularly in South and Southeast Asia (Bridge and Page, 1982; Netscher & Erlan, 1993; Soriano et al., 2000). *M. graminicola* significantly limits the yield of lowland (irrigated), upland (rain-fed), deep-water and aerobic rice (Bridge and Page, 1982; Das et al., 2011; Padgham et al., 2004; Prot and Rahman, 1994; Soriano and Reversat, 2003; Win et al., 2015a). In rice, the degree of susceptibility to and growth damage from *M. graminicola* appears to be closely related to the soil population density of *M. graminicola* (Cabasan et al., 2017; Khan and Ahamad, 2020; Poudyal et al., 2005). Spatiotemporal studies have shown that the densities of *M. graminicola* in the soil and rice roots fluctuate throughout the year (Nagachandrabose et al., 2010; Win et al., 2013). The dynamic changes in nematode populations in agroecosystems are affected by factors such as soil structure, temperature, pH, water regime, growth stage of the host plant, and crop cycle duration, which in turn affect nematode survival and ultimately the ability to infect plants (Cabasan et al., 2017; Jaiswal et al., 2012; Soriano et al., 2000; Win et al., 2013; Win et al., 2015a).

In the past decade, *M. graminicola* has been detected in many provinces in China and has had a severe incidence in Hunan Province (Feng et al., 2017; Ju et al., 2020; Liu et al., 2021; Song et al., 2017; Xie et al., 2019). A full understanding of the population dynamics of *M. graminicola* within and between crop cycles facilitates management decisions and the development of new and/or more accurate management practices (Win et al., 2013). However, the population density dynamics of *M. graminicola* in the soil in different direct-seeded rice agroecosystems in China remain unknown. Hence, an investigation and pot experiment were undertaken to monitor the population dynamics of *M. graminicola* in a rain-fed upland agroecosystem with water direct-seeded rice, a dry aerobic rice agroecosystem with dry direct-seeded rice, and a lowland double-rice cropping sequence agroecosystem with wet direct-seeded rice. The aim of this study was to provide valuable insights into the fluctuations in *M. graminicola* populations within these specific rice cultivation systems.

## Materials and Methods

### Experimental site and sampling design

Three naturally nematode-infested rice fields located in Anding town, Pingjiang County, Yueyang City, Hunan Province (28°57′N, 113°67′E), were selected as the site for our population study (Peng et al., 2022). The laboratory work was performed at the Nematology Laboratory, College of Plant Protection, Hunan Agricultural University. Four *M. graminicola*-susceptible Chinese rice varieties, Huanghuazhan, Hanyou73, Xiangzaoxian45 and Zhuliangyou819, were included in our study.

The first field site represented the rain-fed upland rice agroecosystem. Rice seeds of cv. Huanghuazhan were germinated at 30 °C for 2 days, and the germinated seeds were then sown in nematode-infested paddy fields with fairly shallow (1-2-cm-deep) water by water direct seeding, and cultivated in upland rice culture conditions until maturity (late April to late September).

The second field site represented the dry aerobic rice agroecosystem. Rice seeds of cv. Hanyou73 (a water-saving and drought-resistant hybrid rice) were geminated at 30 °C for 2 days, and the germinated seeds were then sown in nematode-infested paddy fields by dry directed seeding. Water was only added to the field when the average soil water tension fell below −50 kPa, as measured with a tensiometer (Kumar et al., 2008), and when most plants began to wilt and show leaf rolling. This treatment continued until maturity (late April to late September).

The third field site represented the lowland double-rice cropping sequence agroecosystem. Rice seeds of cv. Xiangzaoxian45 were geminated at 30 °C for 2 days, and the germinated seeds were then sown in nematode-infested paddy fields by wet direct seeding and cultivated in conventional lowland irrigated rice until maturity (mid-April to mid-July). Subsequently, the variety Zhuliangyou819 was planted in the same field according to the same germination, sowing and cultivation conditions as Xiangzaoxian45 (late July to late October). At the three paddy field sites, the rice piles were left in the fields after the straw was harvested, and the fields were left fallow during the winter months.

### Assessment of field and environmental conditions

Three field soil-related factors, including soil pH, soil texture and soil organic matter, were assessed at Nanjing WEBiolotech Biotechnology Co., Ltd (Table 1). The ambient air temperature and rainfall throughout the year were obtained from the Pingjiang meteorological station. The annual average air temperature from May 2021 to April 2022 was 18.7 °C. The temperature from May 2021 to September 2021 was highest, then began to drop in October 2021, with the lowest temperature falling below 5 °C in winter. Heavy rain was recorded in May, July and August 2021, while drought was observed in September.

**Table 1:** Characteristics of soil related factors from three rice agroecosystem fields.

**Soil-related factors**		**Rain-fed upland rice agroecosystem**	**Dry aerobic rice agroecosystem**	**Lowland double-rice cropping sequence agroecosystem**
Soil texture class		clay loam	clay loam	sandy loam
Texture %	Sand	20.3	23.3	62.8
	Silt	37.8	42.6	26.1
	Clay	41.9	34.1	11.1
pH		5.00	5.15	5.20
Total nitrogen (g/kg)		0.55	0.71	1.74
Hydrolytic nitrogen (mg/kg)		59.13	77.37	198.42
Total phosphorus (g/kg)		0.36	0.34	0.73
Available phosphorus (mg/kg)		4.54	2.30	10.70
Total potassium (g/kg)		9.67	12.46	14.64
Available potassium (mg/kg)		97.23	125.48	160.28
Organic matter (g/kg)		10.11	14.53	32.71

### Sample collection

Between early May 2021 and late April 2022, soil and root samples were collected every other week during the rice planting period and every month during the fallow period. Rhizosphere soil and the whole root system of three rice hills were collected at five sampling sites in a 1-ha field. Each selected plant was carefully uprooted, rhizosphere soil was collected up to a depth of 20 cm, and the rice shoots were cut off. Soil and root samples were transported to the laboratory immediately after sampling. Soil nematodes were extracted, and root galls were investigated on the day of sampling.

### Assessment of *M. graminicola* population densities in soil

The collected rhizosphere soil samples from each rice field were taken back to the laboratory, mixed, and divided into three subsamples of 100 cm^3^ soil as three replicates. Nematodes were extracted from each 100 cm^3^ soil subsample by the sucrose centrifugation method (Li et al. 2016). The specific operation was as follows: a 100 cm^3^ soil sample was poured into a centrifuge tube, and 200 mL 1.0% NaClO solution was added, fully stirred with a glass rod, and centrifuged at 3,000 r/min for 3 min. The supernatant was successively poured into 40-mesh, 80-mesh and 500-mesh sieves. The nematodes on the 500-mesh sieve were collected after washing with tap water, and the number of nematodes was counted under a microscope. At the same time, 200 mL sucrose solution with a density of 1.18 was added to the precipitate, fully stirred with a glass rod, and centrifuged at 3000 r/min for 3 min. The supernatant was successively screened through 40-mesh, 80-mesh and 500-mesh sieves, the nematodes on the 500-mesh sieve were collected, and the separated J2 and eggs were counted under a microscope.

### Assessment of galling severity

The roots of each rice plant were washed free of soil with tap water and scored for infection severity using the gall index according to Zeck (1971). Root galling was rated on Zeck’s scale of 0 to 10, where 0 = no galls, 1 = very few small galls, 2 = numerous small galls, 3 = numerous small galls, of which some have grown together, 4 = numerous small and some large galls, 5 = 25% of roots severely galled, 6 = 50% of roots severely galled, 7 = 75% of roots severely galled, 8 = no healthy roots but plant is still green, 9 = roots rotting and plant dying, and 10 = plant and roots dead. The gall index (GI) was calculated using the formula $\mathrm{GI}=\frac{\Sigma \left(\mathrm{Si}\times \mathrm{Ni}\right)}{N\times 10}\times 100$ , where S_i_ = root galling scale of 0 to 10, N_i_ = the number of plants in each root galling scale, and N = total number of plants examined.

### Pot experiment and Pluronic gel assay

Soil with *M. graminicola* eggs and J2 was obtained from a heavily infected rice nursery in a rice field in Pingjiang County, Yueyang city, Hunan Province (28°57′N, 113°67′E). The population densities of *M. graminicola* eggs and J2 in soil were measured as described above. The numbers of *M. graminicola* eggs and J2 in the soil were approximately 200±23 and 300±52 per 100 cm^3^ of soil, respectively. One-week-old seedlings (cv. Zhuliangyou35) were transplanted into a plastic pot (21.5-cm-deep and 23-cm-diam.) containing 5,000 cm^3^ of the soil with the *M. graminicola* eggs and J2. The soil moisture was maintained at 70–90% for one week after rice seedling transplantation, and then the water treatment was applied.

Water management of the potted plants was divided into three types over the period of the experiment: the first type was the upland mode, the second type was the semi-submerged mode, and the third type was the flooded mode. All the treatments were replicated 21 times in a randomized complete block design, and plants were grown from May through July 2022 under greenhouse conditions. After one month, the number of eggs and J2 in the soil and rice roots were investigated, and three pots were also investigated each time for each water management treatment. The eggs and J2 populations in the soil and root tissues were extracted using the aforementioned method and the method detailed by Hussey and Barker (1973), respectively.

The Pluronic gel assay was performed according to Wang et al. (2009), and 23 g Pluronic F-127 powder (PF-127 Sigma-Aldrich) was fully dissolved in 100 mL of distilled water, with stirring for 24 h at 4 °C. One-week-old seedlings (cv. Zhuliangyou35) were transplanted into an acrylic tube with 10 ml PF-127 gel, and approximately 100 hatched J2 were added. The development of nematodes was observed under a microscope.

### Data analysis

Statistical analyses were performed with the Data Processing System (DPS) statistical software package (Tang & Zhang, 2013). ANOVA, followed by Duncan’s multiple range test, was used to evaluate the significant effects of the treatments at a significance level of *P* < 0.05.

## Results

### Effects of different types of rice agroecosystems on *M. graminicola* population density in soil

**Figure 1:**
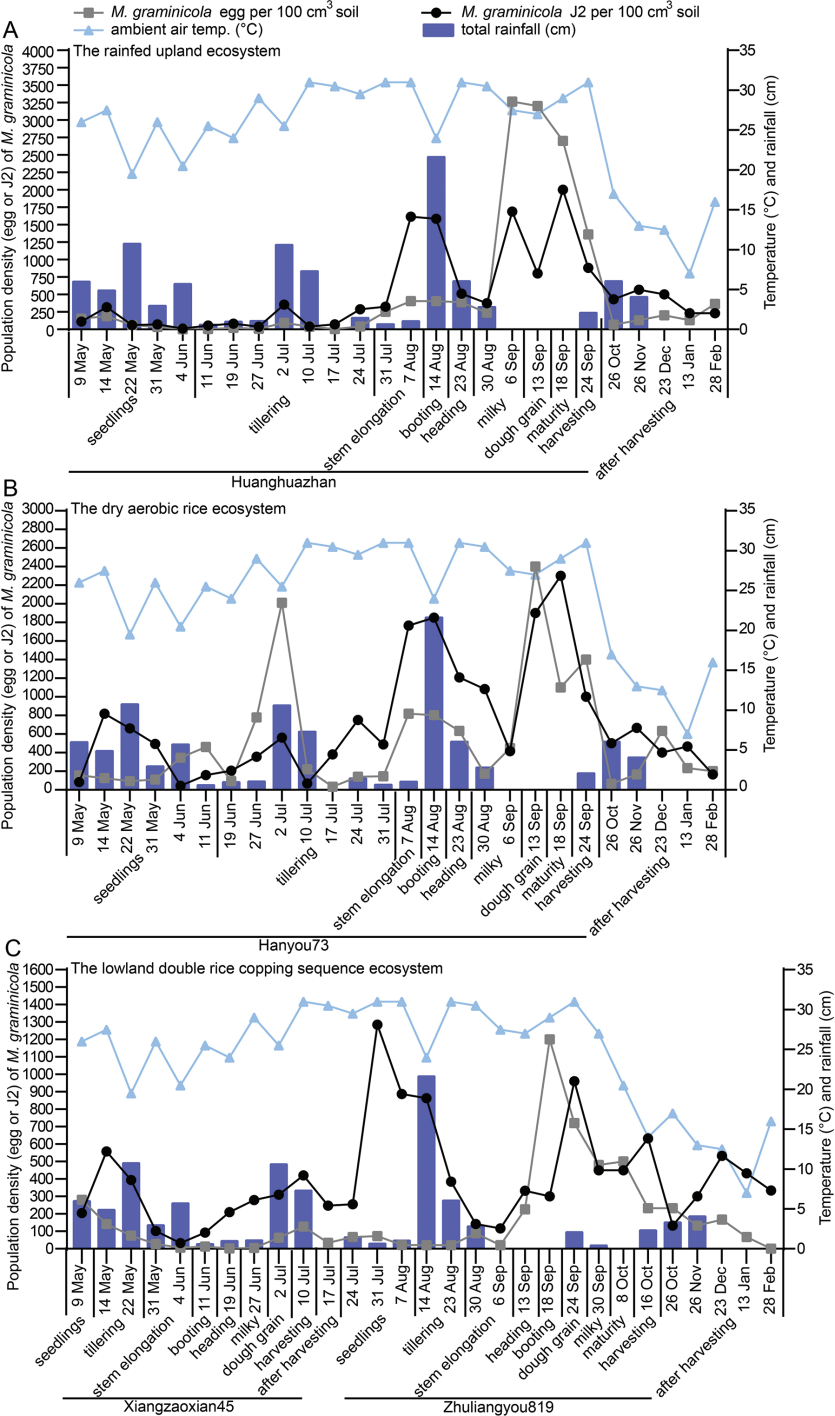
Population dynamics of *M. graminicola* eggs and second-stage juveniles (J2) in the soil in the rain-fed upland agroecosystem (A), the dry aerobic rice agroecosystem (B) and the lowland double-rice cropping sequence agroecosystem (C) from May 2021 to February 2022. The initial *M. graminicola* populations in the soil in the rain-fed upland agroecosystem, the dry aerobic rice agroecosystem and the lowland double-rice cropping sequence agroecosystem were 102 ± 32 J2s and 36 ± 8 eggs/100 cm^3^ soil, 467 ± 102 J2 and 113 ± 38 eggs/100 cm^3^ soil, and 246 ± 64 J2s and 52 ± 17 eggs/100 cm^3^ soil, respectively.

The population densities of *M. graminicola* were significantly affected by the three different rice cultivation systems and the environmental conditions (Fig. 1). The population density of the *M. graminicola* J2 and eggs in the soil was significantly higher in the dry aerobic rice agroecosystem and in the rain-fed upland agroecosystem than in the lowland double-rice cropping sequence agroecosystem. In all three systems, the nematode density appeared to peak in September, when the rainfall was low, and decreased when the temperature fell after the rice harvest.

In the rain-fed upland rice agroecosystem, the population density of *M. graminicola* in the soil showed two distinct peaks, which occurring from August 7 to August 14 and September 6 to September 18 (Fig. 1A). The lowest population density was observed at the seedling stage (202 eggs + J2 per 100 cm^3^ soil) and the tillering stage (164 eggs + J2 per 100 cm^3^ soil) of the rice plant. With the decrease in rainfall at the end of July, the number of rice root galls increased, and the population density of the nematodes in the soil gradually increased. The population density reached its first peak at the stem elongation stage (2020 eggs + J2 per 100 cm^3^ soil) of the rice plant. Then, with heavy rainfall in mid- August, the population density gradually decreased at the heading stage (898 eggs + J2 per 100 cm^3^ soil) and early milky stage (615 eggs + J2 per 100 cm^3^ soil). A sharp rise in the density of nematodes at the late milky stage (4951 eggs + J2 per 100 cm^3^ soil) marked the second peak, with low rainfall from early to mid-September. Afterward, the nematode density continued to decline with decreasing air temperature until after harvesting (573 eggs + J2 per 100 cm^3^ soil).

In the dry aerobic rice agroecosystem, the population dynamics of *M. graminicola* in the soil showed three peaks (Fig. 1B), including one at the tillering stage (2571 eggs + J2 per 100 cm^3^ soil), booting stage (2655 eggs + J2 per 100 cm^3^ soil) and the dough grain stage (4300 eggs + J2 per 100 cm^3^ soil). The first two peaks occurred when the rainfall was high, while the final peak occurred when the rainfall was low. From the beginning of May to the middle of June, the population density of *M. graminicola* in soil was at a low level and gradually increased in late June, reaching the first peak at the tillering stage on July 2. After that, its population density gradually decreased and remained at a low level from June 10 to the end of July, and increased gradually to the second peak during the booting stage in the middle of August. At the beginning of September, the population density in the soil increased gradually and reached a third peak at the dough grain stage on September 13. After harvesting, the population density in the soil was at a low level as the air temperature decreased.

In the lowland double-rice cropping sequence agroecosystem, the population density of *M. graminicola* had two peaks in each season (Fig. 1C). In the second season, the population density of *M. graminicola* in the soil was significantly greater than the first season at the seedling, booting and dough grain stages. The two peaks in the first season occurred when the rainfall was relatively high, while the two peaks in the second season occurred when the rainfall was low. During the first season, the population density of *M. graminicola* in the soil showed two distinct peaks at the tillering stage (700 eggs + J2 per 100 cm^3^ soil) and harvesting stage (546 eggs + J2 per 100 cm^3^ soil). There were two peaks of nematode density in the second season, which occurred at the seedling stage (1358 eggs + J2 per 100 cm^3^ soil) and dough grain stage (1680 eggs + J2 per 100 cm^3^ soil). Similarly to the previous study site, the soil nematode population density gradually declined with decreasing air temperature after harvesting (467 eggs + J2 per 100 cm^3^ soil).

### Effect of different types of rice agroecosystems on rice root gall and gall index

**Figure 2:**
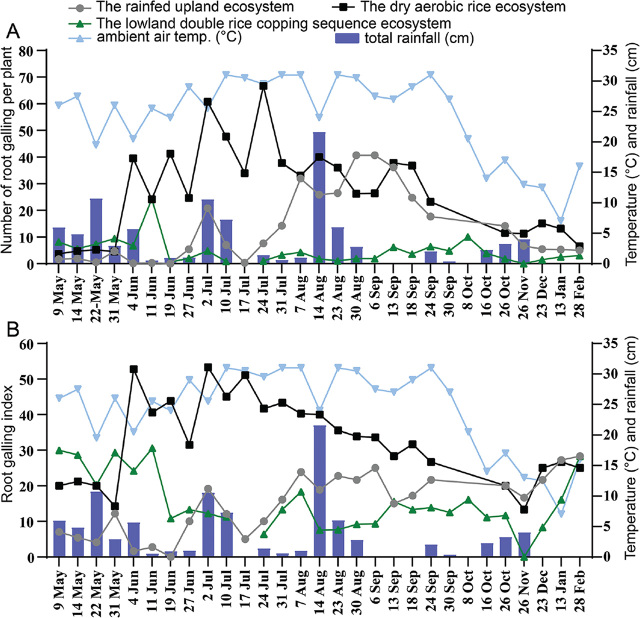
Fluctuations in root galling (A) and root galling index (B) caused by *M. graminicola* recorded on rice roots in the rain-fed upland agroecosystem (variety Huanghuazhan), the dry aerobic rice agroecosystem (variety Hanyou73) and the lowland double-rice cropping sequence agroecosystem (variety Xiangzaoxian45 and Zhuliangyou819) from May 2021 to February 2022.

Both the root gall induced by *M. graminicola* and the root gall indices were markedly influenced by the three different rice cultivation systems (Fig. 2). Roots in the dry aerobic rice agroecosystem developed more root galls than those in the rain-fed upland agroecosystem and in the lowland double-rice cropping sequence agroecosystem. The number of root galls remained at low levels in the lowland double-rice cropping sequence agroecosystem. Similar trends were observed for the root gall index in the three rice cultivation systems.

In the rain-fed upland agroecosystem, the number of root galls remained at low levels during the vegetative growth stage, including the seedling, tillering and stem elongation stages, while the number of root galls in the reproductive growth stage was high. After the rice harvest, the number of root galls decreased gradually and declined to five root galls per plant at one month after harvest. In the dry aerobic rice agroecosystem, the number of root galls was relatively low at the seedling stage, but remained high at the tillering stage until harvest, with up to 67 root galls per plant at the tillering stage. In the lowland double-rice cropping sequence agroecosystem, the highest root gall number occurred at the booting stage (24 root galls per plant) in the first season, while it remained at low levels throughout the second season.

The root gall indices in the dry aerobic rice agroecosystem were significantly higher than those in the rain-fed upland agroecosystem and the lowland double-rice cropping sequence agroecosystem (Fig. 2B). The maximum root gall indices were 23.8 at the stem elongation stage in the rainfed upland agroecosystem, 53.3 at the seedling stage in the dry aerobic rice agroecosystem and 30.6 at the booting stage in the first season of the dry aerobic rice agroecosystem. The root-knot index of the rain-fed upland agroecosystem was at a lower level during the seedling stage to tillering stage, and then increased gradually until harvest. The early seedling stage root gall index in the dry aerobic rice agroecosystem was low, but increased to a higher level from the late seedling stage to the tillering stage, and then gradually decreased until after harvest. In the lowland double-rice cropping sequence agroecosystem, the root gall indices were highest at the seedling stage and tillering stage during both seasons, and then decreased gradually with the growth of the rice.

**Figure 3:**
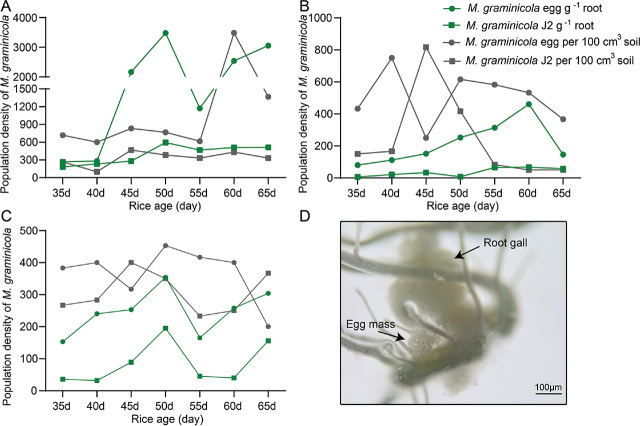
Zhuliangyou35 infected with *M. graminicola*. Population dynamics of *M. graminicola* eggs and second-stage juveniles (J2) in the soil and rice roots in the pot experiments under upland (A), semi-submerged (B) and flooded (C) conditions. (D) Microscopic observations of egg masses in the PF-127 gel.

Water regime effects on the population density of *M. graminicola*: The results from the three agricultural systems showed that rainfall affected soil nematode population density. To verify the relationship between soil moisture and nematode density, we conducted a pot experiment with a water treatment (Figs. 3 A–C). The population density of *M. graminicola* in the soil and rice roots in the upland mode was significantly higher than that in the semi-submerged and flooded modes, which indicated that low soil moisture was conducive to the reproduction of *M. graminicola*. The nematode density in the semi-submerged mode was higher than that in the flooded mode, while the nematode density in the rice roots was the opposite. The soil’s nematode population in the flooded mode was the lowest among the pot experiments. In addition, in our laboratory experiments, *M. graminicola* was found to lay eggs outside the roots of rice (Fig. 3D).

Based on the above results, we propose a hypothesis that *M. graminicola* lays egg masses within roots under flooding conditions and might tend to lay egg masses outside the roots under suitable soil moisture conditions. Therefore, the nematode density in the soil increases with suitable soil moisture in low rainfall or upland agroecosystems.

## Discussion

With the progress of agricultural modernization, the simplification and mechanization of farming have become inevitable. The global popularization and application of direct-seeding rice cultivation technology has a wide and profound impact on the occurrence of rice diseases, insect pests and weeds (Farooq et al., 2011; Chaudhary et al., 2023). Thus, it was of practical significance to investigate the effects of different types of direct-seeded rice agroecosystems on the population dynamics of root-knot nematodes, as this can provide theoretical guidance for the control of root knot nematodes in direct-seeded rice cultivation technology.

The dynamics of nematode populations are closely related to the rice cultivation model employed. Our study revealed that in the dry aerobic rice agroecosystem, the population density of *M. graminicola* J2 and eggs in the soil, as well as the presence of root galls and root gall index, were higher than those in the rain-fed upland agroecosystem and the lowland double-rice cropping sequence agroecosystem. These results indicated that the dry aerobic rice agroecosystem provides more favorable conditions for the invasion and propagation of *M. graminicola*, which is consistent with the results of previous reports (Das et al., 2011; De Waele et al., 2013; Soriano et al., 2000). We also observed that in the second season, the number of root galls and root gall index were lower than those of the first season in the lowland double-rice cropping sequence agroecosystem. This may be attributed to the fast and deep growth of roots during the seedling stage of the second rice season, influenced by the high temperature in July. Such conditions may not favor nematode colonization. Hence, more attention should be given to the control of root knot nematodes in the seedling stage of the first season of double-cropping rice planting.

The water regime is a key environmental factor in the survival of *M. graminicola*, and since plant-parasitic nematodes are aquatic animals, their development needs to occur surrounded by a water film (Simons, 1973). In this study, the population of nematodes was from May to July was significantly lower in the rain-fed upland agroecosystem with water direct-seeded rice than in the dry aerobic rice agroecosystem with dry direct-seeded rice or in the lowland double-rice cropping sequence agroecosystem with wet direct-seeded rice. These results suggested that direct water seeding could effectively prevent the invasion of nematodes into rice seedlings, thereby reducing their reproduction. This is consistent with other investigations indicating that flooded conditions can inhibit the invasion of nematodes into rice roots (Cabasan et al., 2017; De Waele and Elsen, 2007; Win et al., 2015b). Therefore, proper water management during the sowing period of direct-seeding rice is essential for nematode control. Rainfall is a key factor affecting soil moisture in the field, and low rainfall in September creates favorable conditions for *M. graminicola* to penetrate rice roots, resulting in an increase in nematode population density in the soil across the three agroecosystems. Conversely, the continuous rainfall in early July led to a gradual decrease in nematode population density in both the rain-fed upland agroecosystem and the dry aerobic rice agroecosystem. In the lowland double-rice cropping sequence agroecosystem under conventional lowland irrigated rice cultivation, the flooded paddy field environment maintained the number of root galls at low levels throughout both seasons. These results indicated that field water retention can inhibit nematode infection and thus hinder nematode propagation. Similar to the findings of Garg et al. (1995), prolonged early flooding reduces soil aeration, limits nematode respiration and movement, and ultimately decreases the population of *M. graminicola*. These results were consistent with previous findings that the water regime affects the prevalence of *M. graminicola* and the degree of damage to rice (Cabasan et al., 2017; Tandingan et al., 1994; Win et al., 2015b).

Temperature is an important factor affecting nematode infection, survival, and life cycle (Velloso et al., 2022). In the three agroecosystems, the decrease in temperature and the rice harvest, the nematode population density in the soil gradually decreased, and the number of rice root galls also gradually decreased.

The results of our pot experiment were consistent with those of the paddy field investigation, both of which prove that field water is closely related to the reproduction of nematodes and that low soil moisture is conducive to the reproduction of nematodes. The importance of the water regime to nematodes has also been illustrated in several other reports (Cabasan et al., 2017; Nzogela et al., 2019; Soriano et al., 2000; Win et al., 2013). Previous studies have reported that nematodes of *M. graminicola* lay eggs inside the root cortex, and hatched J2 can be released into the soil or remain within the gall to migrate and reinfect the same or adjacent roots by establishing new feeding sites (Mattos et al., 2019; Kyndt et al., 2012; Rao and Israel, 1973). However, in our laboratory experiments, we found that *M. graminicola* lay eggs not only inside the roots, but also outside the roots. This result was consistent with that of Khan et al. (2004), who reported an egg mass on the root surface in some species of weeds. In conjunction with our results showing a high population density of *M. graminicola* eggs in soil at suitable soil moisture levels, we propose a hypothesis that nematodes lay eggs inside roots under flooding conditions and might lay eggs outside roots when the soil moisture is suitable.

Our study showed that three agroecosystems – the rain-fed upland agroecosystem, the dry aerobic rice agroecosystem and the lowland double-rice cropping sequence agroecosystem – supported the survival of *M. graminicola*. Field moisture was a key factor affecting nematode population dynamics, followed by temperature. The population density of *M. graminicola* was significantly greater in the dry aerobic rice agroecosystem with low soil moisture than in the rain-fed upland agroecosystem or the lowland double-rice cropping sequence agroecosystem. In addition, we found that nematodes might lay eggs outside roots when the soil moisture is suitable. This study provides a reference for understanding the population dynamics of rice root-knot nematodes in direct-seeded rice agricultural systems, which is beneficial for their control.
